# Which Stage of ADPKD Is More Appropriate for Decortication? A Retrospective Study of 137 Patients from a Single Clinic

**DOI:** 10.1371/journal.pone.0120696

**Published:** 2015-05-04

**Authors:** Xiaoqiang Qian, Xujun Sheng, Ruipeng Li, Hailong Liu, Xiangjie Kong, Liujian Duan, Jun Qi

**Affiliations:** Department of Urology, Xinhua Hospital, Shanghai Jiaotong University School of Medicine, Shanghai, China; Sun Yat-sen University, CHINA

## Abstract

**Objective:**

To study retrospectively the efficacy of decortication in patients with different stages of ADPKD and to determine which stage for decortication is more appropriate.

**Materials and Methods:**

We analyzed 137 patients with ADPKD from 2001 to 2010. All patients were divided into three stages. A total of 70 patients underwent decortication, and we studied intraoperative indicators and postoperative indicators at 1 and 3 years follow-up.

**Results:**

In 70 patients who underwent decortication, significant differences were observed in operative duration and bleeding volume between patients with stage I and II ADPKD (P<0.05), but no significant differences were observed in intestinal recovery time, pain medication dose, and the days of postoperative hospitalization (P > 0.05). The total complication occurrence rate was significantly different between them (P < 0.05). The serum creatinine (Scr) levels in patients with stage I ADPKD were within normal limits 1 and 3 years postoperatively and did not differ significantly (P > 0.05). Scr levels were significantly decreased in patients with stage II ADPKD in the 1st postoperative year (P < 0.05), but these were not significant differences in the 3rd postoperative year (P > 0.05). In the 1st postoperative year, VAS value, blood pressure and renal volume significantly differed (P < 0.05). However, no significant differences were observed 3 years later (P > 0.05).

**Conclusions:**

Decortication in patients with stage I ADPKD can alleviate back pain symptoms and decrease blood pressure within 1 year, but the long-term efficacy is not ideal. Scr levels can be maintained within normal limits, suggesting that decortication does not lead to deterioration of renal function. For patients with stage II ADPKD, decortication can significantly improve renal function over the short term. However, after 3 years, renal function returns to the preoperative level, and surgical difficulties and complications also increase.

## Background

Autosomal dominant polycystic kidney disease (ADPKD), a progressive hereditary disorder, is characterized by formation of bilateral multiple cysts of the kidneys. Its incidence is 1:400–1:1,000[1]). A total of 10% of patients with hemodialysis (HD) and 5% of patients with renal transplantation suffer from it[2]. The conventional disposal is to exclude the cyst infection and to treat patients conservatively. These patients may be offered percutaneous, laparoscopic or open surgical cyst decortication after failed conservative therapy[3]. We retrospectively analyzed our clinical data of 137 patients with ADPKD from 2001 to 2010. Among 137 patients, 70 patients with stage I and II ADPKD underwent decortication. By comparing intraoperative and postoperative indicators, we evaluated the efficacy of decortication in the treatment of patients with stage I and II ADPKD. Our findings may help physicians decide which patients of ADPKD are more appropriate for decortication.

### Materials

From Feb 2001 to Sept 2010, 137 patients with ADPKD were admitted to Xinhua hospital. All of them were confirmed by B ultrasound or computed tomography (CT). Follow-up periods ranged from 1 to 10 years, and the average duration was 5.2±1.4 years. We explored and created a clinical cut-off for ADPKD stage according to mainly serum creatinine (Scr) levels and blood pressure (BP) values. In stage I, Scr levels and BP values were within normal limits. In stage II, Scr levels and BP values were mildly abnormal (Scr < 200 μmol/L, BP < 150/100 mmHg). In stage III, Scr levels and BP values were significantly abnormal (Scr ≥ 200 μmol/L, BP ≥ 150/100 mmHg). Among 137 patients, 70 with stage I and II ADPKD underwent open decortication, and their preoperative general data are shown in Table 1.

**Table 1:** Preoperative general data of 70 patients.

	Stage I	Stage II	P
Patients (male/female)	43 (16/27)	27 (11/26)	>0.05
Age (years)	44.1 ± 7.9	51.4 ± 9.0	<0.05
Systolic BP (mmHg)	123.0 ± 11.2	142.0 ± 3.8	<0.05
Diastolic BP (mmHg)	81.0 ± 3.5	95.0 ± 3.1	<0.05
Scr (μmol/L)	81.0 ± 11.3	152.0 ± 20.3	<0.05

## Methods

### 1. Preoperative preparations

We regularly examined blood routine, urine routine, and coagulation function tests; evaluated the function of important organs, including the heart, lung, liver and cerebrum; recorded BP values and renal function (Scr levels and glomerular filtration rate); and examined the abdomen with B ultrasound and plain CT.

### 2. Decortication

All patients were placed in a lateral position while under general anesthesia, and a lumbar incision was made. We opened the Gerota fascia under the 11th or 12th rib. After isolating the kidney completely and conducting renal denervation, we decorticated all the renal cysts that were in sight. As for the large cysts, we isolated almost all the parts of the cysts, opened the cyst walls, and suctioned the cyst fluid. We resected the cyst wall 5 mm lateral to the renal parenchyma and stopped the bleeding completely. As for the small cysts, we used an ultrasonic scalpel to resect the cyst wall and then suctioned the cyst fluid. Perioperatively, we used ice flakes to keep the renal area cold.

### 3. Observed indicators

#### 1) Perioperative period

(1)operative duration, (2)bleeding volume, (3)postoperative intestinal recovery time, (4)postoperative pain medication dose, (5)days of postoperative hospitalization, and(6)postoperative complications (urine leakage, intestinal adhesion and obstruction, retroperitoneal hematoma).

#### 2) Follow-up from 1 to 10 years

(1)renal function (Scr level), (2)BP value (systolic/diastolic blood pressure), (3)VAS (Visual Analogue Scale) value and(4)renal volume.

### 4. Statistical analysis

We used SPSS 17.0 software to analyze data, and all data were shown as mean ± standard deviation (SD). Chi-square and Fisher’s exact tests were used to analyze categorical variables, and t-tests were used for continuous variables. All results were considered to be significant with P values < 0.05.

## Results

### 1. Comparison of perioperative and operative data between patients with stage I and II ADPKD (Table 2)

**Table 2:** Comparison of the perioperative and operative data between patients with stage I and II ADPKD.

Stage	OD (min)	BV (mL)	IRT (days)	PMD (mg)	DPH (days)
I	76.0 ± 5.3	49.0 ± 11.2	1.6 ± 0.5	100.0 ± 13.2	7.0 ± 2.4
II	113.0 ± 9.8	107.0 ± 23.1	1.8 ± 0.7	107.0 ± 23.1	7.9±2.3
P	<0.05	<0.05	>0.05	>0.05	>0.05

OD: Operative duration; BV: Bleeding volume; IRT: Intestinal recovery time; PMD: Pain medication dose; DPH: Days of postoperative hospitalization

Among all patients with ADPKD who underwent decortication, operative duration in patients with stage I ADPKD was 60–90 min (76.0 ± 5.3 min), bleeding volume was 20–80 mL (49.0 ± 11.2 mL), intestinal recovery time was 1–3 days (1.6 ± 0.5 days), pain medication (Bucinnazine) dose was 50–150 mg (100.0 ± 13.2 mg), and the days of postoperative hospitalization were 5–9 days (7.0 ± 2.4 days). Operative duration of patients with stage II ADPKD was 100–160 min (113.0 ± 9.8 min), bleeding volume was 75–200 mL (107.0 ± 23.1 mL), intestinal recovery time was 1–5 days (1.8 ± 0.7 days), pain medication (Bucinnazine) dose was 50–200 mg (107.0 ± 23.1 mg), and the days of postoperative hospitalization were 5–10 days (8.0 ± 1.3 days). Significant differences were observed in operative duration and bleeding volume between patients with stage I and II ADPKD (P < 0.05). No significant differences were observed in intestinal recovery time, pain medication (Bucinnazine) dose, and the days of postoperative hospitalization between patients with stage I and II ADPKD (P > 0.05).

### 2. Comparison of decortication complications between patients with stage I and II ADPKD (Table 3)

**Table 3:** Comparison of decortication complications between patients with stage I and II ADPKD.

Stage (cases)	IA	IO	UL	ACI	RH	TC
I (n = 43)	2 (4.7%)	1 (2.3%)	3 (6.9%)	2 (4.7%)	1 (2.3%)	9 (20.7%)
II (n = 27)	3 (11.1%)	2 (7.4%)	4 (14.8%)	4 (14.8%)	1 (1.1%)	14 (51.8%)
P	>0.05	>0.05	>0.05	>0.05	>0.05	<0.05

IA: Intestinal adhesion; IO: Intestinal obstruction; UL: Urine leakage; ACI: Abdominal cavity infection; RH: Retroperitoneal hematoma; TC: Total complications

Postoperative complications in 70 patients mainly included intestinal adhesion(I: 2/43, II: 3/27), intestinal obstruction (I: 1/43, II: 2/27), urine leakage (I: 3/43, II: 4/27), abdominal cavity infection (I: 2/43, II: 4/27), and retroperitoneal hematoma (I: 1/43, II: 1/27). No significant differences were observed in these complications between patients with stage I and II ADPKD (P > 0.05). However, the total complications’ occurrence rate (I: 9/43, II: 14/27) differed significantly (P < 0.05).

### 3. Comparison of clinical efficacy over 3 years between patients with stage I and II ADPKD (Table 4)

**Table 4:** Comparison of decortication efficacy on the 1^st^ and 3^rd^ year between patients with stage I and II ADPKD.

SF	Scr (μmol/L)	DBP (mmHg)	SBP (mmHg)	RV (mL)	VAS
I preoper	81.0 ± 11.3	123.0 ± 11.2	81.0 ± 3.5	492 ± 208	3.0 ± 1.2
1st year	78.0 ± 8.1^b^	114.0 ± 3.8^a^	65.0 ± 7.6 ^a^	392 ± 132 ^a^	1.0 ± 0.2 ^a^
3rd year	82.0 ± 10.2^b^	125.0 ± 9.1 ^b^	82.0 ± 2.7 ^b^	504 ± 145 ^b^	3.0 ± 0.5 ^b^
II preoper	152.0 ± 20.3	142.0 ± 3.8	95.0 ± 3.1	642 ± 204	6.0 ± 2.4
1st year	102.0 ± 4.3^a^	118.0 ± 4.8 ^a^	80.0 ± 8.7 ^a^	403 ± 162 ^a^	3.0 ± 1.7 ^a^
3rd year	154.0±10.4^b^	144.0 ±6.8 ^b^	93.0 ± 7.3 ^b^	710 ± 125 ^b^	7.0 ± 1.9 ^b^

SF: Stage and follow-up; DBP: Diastolic Blood Pressure; SBP: Systolic Blood Pressure; RV: Renal volume;VAS: Visual Analogue Scale

Compared with the 1st-year follow-up,

^a^P < 0.05; Compared to the 3rd-year follow-up,

^b^P > 0.05

In patients with stage I ADPKD, Scr levels after 1–3 years of follow-up were within normal limits, and no significant difference was observed between these levels and the preoperative levels (P > 0.05). In one year, the postoperative diastolic and systolic BP, renal volume, and the VAS value were significantly different from the preoperative value (P < 0.05). However, no significant differences were observed in the values 3 years postoperatively (P > 0.05). In patients with stage II ADPKD, Scr levels, diastolic and systolic BP, renal volume, and VAS values were significantly different after 1 year of follow-up (P < 0.05). However, no differences were observed after 3 years of follow-up (P > 0.05).

## Discussion

Of 70 patients who underwent decortication, the pathology perioperatively and postoperatively showed that the renal cortex and medulla had multiple fluid-filled cysts that had formed and were enlarged along with kidney tubule mesenchymal inflammation, collagen fibrosis and augmented cell quantities. The characteristic pathological features of ADPKD included cystic lesions in the kidneys, liver, pancreas and arachnoid. Other extrarenal complications included colonic diverticula, intracranial aneurysms, aortic aneurysms and valve abnormalities[4]. In clinical practice, we used the following B ultrasound criterion of Ravine to diagnose ADPKD[5]: age < 30 years, two unilateral or bilateral cysts of the kidney; age:30–59 years, ≥3 unilateral cysts of the kidneys; and age ≥ 60 years, ≥4 unilateral cysts of the kidneys.

Surgery indications for ADPKD had been disputed for a long period. After comprehensive reviews of data[6–11], we determined the following indications including Scr < 200 μmol/L, renal function that was normal or mildly abnormal, BP < 180/100 mmHg, which could be controlled within normal limits with the use of high blood pressure drugs, B ultrasound or CT examination indicated that the collective system was pressured and the maximal cyst was >4 cm, recurrent urinal tract infection that was possibly accompanied by calculi with no improvement with conservative treatment, frequent or sustainable back pain with no improvement with conservative treatment, and suspected renal tumor. In addition, some articles[6] reported that bilateral decortication was more effective than unilateral decortication. We tended to carry out the bilateral operation by two steps, and the first step was prior to the severer side and the larger volume cyst of the polycystic kidney.

There were also studies[7–9] shown that decortication might not prolong the augmentation of renal cysts with respect to the beginning and the development mechanisms of the cyst. In contrast, after the perirenal fascia was cut, the large renal cysts were decorticated, and the visualized cysts were depressurized; thus, countless microcysts might lose their original constraints and be exposed to a looser growing space. Then, the small cysts might be able to become larger rapidly. Thus, decortication surgery could not prevent the growing of the polycystic kidney. Surgery itself might aggravate renal function and worsen the disease. In view of this, we did not perform surgery in patients with stage III ADPKD. In addition, other contraindications were present, such as blood system diseases and serious cardiac or lung and liver diseases.

So as for patients with stage I and II ADPKD, the VAS value, diastolic and systolic BP, and enlarged renal volume returned to within normal limits significantly (P < 0.05) after 1 year of follow-up. However, in the 3rd year postoperatively, no significant differences were observed compared with those preperatively. Patients with stage I ADPKD might maintain normal renal function after 3 years, but their renal function was normal preperatively. Thus, improving renal function seemed not effective compared with improving high BP and VAS values. Forty-three patients with stage I ADPKD maintained their normal or mildly abnormal renal function after a maximum follow-up of 10 years, suggesting that decortication itself did not lead to deterioration of renal function and might actually protect renal function from entering stage II and III and prolong the patient.

As for patients with stage II ADPKD, their renal function was already mildly abnormal and irreversibly preoperatively. Scr levels could be obviously decreased in the 1st year. However, their Scr levels returned to the original level after 3 years of follow-up. Because the mean age of patients with stage II ADPKD was about 7 years older than that of patients with stage I ADPKD, results of our study suggested that decortication was more appropriate in stage II to improve renal function. However, surgical difficulties and risks (operative duration, bleeding volume, etc.) were increasing, and it was easy to show more postoperative complications. Of 27 patients with stage II ADPKD, 9 (33.3%) had to undergo renal replacement therapy (peritoneal dialysis, HD, or renal transplantation) after their renal function deteriorated. However, the remaining 18 (66.7%) kept their renal function rising up slowly, or they only had to undergo drug therapy 3–5 years postoperatively.

Some patients with stage II ADPKD were observed who were in their 30s with abnormal renal function. Decortication did not prolong the deterioration of renal function, and these patients rapidly entered stage III and had to have replacement therapy. This might be due to the genotype of ADPKD[10].

It was proposed[11] that surgical isolation and division of the periarterial nervous tissue interrupted the sensory pathways from the kidney and limited renal pain. Thus, we carried out to isolate the kidney completely and perform renal denervation.

Postoperative complications in 70 patients mainly included intestinal adhesion (5 patients, 7.1%), intestinal obstruction (3 patients, 4.3%), urine leakage (7 patients, 10.0%), abdominal cavity infection (6 patients, 8.6%), and retroperitoneal hematoma (2 patients, 2.9%). As for urine leakage, most patients could be treated by prolonging drainage alone, but a double-J stent was needed occasionally. Other complications, including intestinal adhesion, intestinal obstruction, abdominal cavity infection, and retroperitoneal hematoma, could be cured by antibiotics and transfusions mostly. As for the recurrent renal cysts, a puncture could ease back pain symptoms momentarily. Open surgery could easily result in infection and hematoma around the kidney. Sometimes we had to remove the involved kidney. Occasionally, the accompanying hepatic cysts were infected and needed drainage or resection.

## Conclusions

Decortication in patients with stage I ADPKD can alleviate back pain symptoms and decrease BP values within 1 year, but the long-term efficacy(over >3 years) is not ideal. Scr levels 1 and 3 years postoperatively can be maintained within normal limits, suggesting that decortication does not lead to deterioration of renal function. For patients with stage II ADPKD, decortication can significantly improve renal function over the short term of 1 year. However, renal function after 3 years returns to the preoperative level, and surgical difficulties and postoperative complications also increase.

## The ethical statement

Ethics Committee of Xinhua Hospital Affiliated to Shanghai Jiaotong University School of Medicine has reviewed the study design and process as well as the applying form,and certifies that this study did not raise any issues of patients’ risk, it was in accordance with the Declaration of Helsinki and was conducted without ethics problems.
